# Altered Insular Function during Aberrant Salience Processing in Relation to the Severity of Psychotic Symptoms

**DOI:** 10.3389/fpsyt.2016.00189

**Published:** 2016-11-23

**Authors:** Anna Walter, Claudia Suenderhauf, Renata Smieskova, Claudia Lenz, Fabienne Harrisberger, André Schmidt, Tobias Vogel, Undine E. Lang, Anita Riecher-Rössler, Anne Eckert, Stefan Borgwardt

**Affiliations:** ^1^Department of Psychiatry (UPK), University of Basel, Basel, Switzerland

**Keywords:** brain imaging, psychosis, salience network, antipsychotic medication, psychopathology, schizophrenia, insula

## Abstract

There is strong evidence for abnormal salience processing in patients with psychotic experiences. In particular, there are indications that the degree of aberrant salience processing increases with the severity of positive symptoms. The aim of the present study was to elucidate this relationship by means of brain imaging. Functional magnetic resonance imaging was acquired to assess hemodynamic responses during the Salience Attribution Test, a paradigm for reaction time that measures aberrant salience to irrelevant stimulus features. We included 42 patients who were diagnosed as having a psychotic disorder and divided them into two groups according to the severity of their positive symptoms. Whole brain analysis was performed using Statistical Parametric Mapping. We found no significant behavioral differences with respect to task performance. Patients with more positive symptoms showed increased hemodynamic responses in the left insula corresponding to aberrant salience than in patients with less positive symptoms. In addition, left insula activation correlated negatively with cumulative antipsychotic medication. Aberrant salience processing in the insula may be increased in psychosis, depending on the severity of positive symptoms. This study indicates that clinically similar psychosis manifestations share the same functional characteristics. In addition, our results suggest that antipsychotic medication can modulate insular function.

## Introduction

Disrupted salience processing has been found to be a principle feature in psychosis (1). Salience is defined as the effectiveness of a stimulus to stand out from its neighbors. A stimulus might be considered to be salient by feature contrast, novelty, emotional, or motivational association (2). The integration of stimuli requires specific brain networks, which associate external stimuli with internal context, thus marking objects that require further consideration (3, 4).

Abnormal salience processing in patients with schizophrenic psychoses seems to arise from inappropriate evaluation of stimuli that would naturally be considered irrelevant (5). Hence, subthreshold stimuli become inappropriately attention-grabbing (6), which is then called aberrant salience. Adaptive motivational salience, by contrast, refers to stimuli with a reliable association with reinforcement and which can therefore influence behavior and attract attention (7). Roiser et al. provided evidence of increases in behavioral aberrant salience in patients with schizophrenia, depending on the severity of their symptoms, primarily with respect to delusions (7).

The neurobiological foundation of the salience network appears to lie in the insula, the anterior cingulate cortex (8, 9), subcortical regions such as the ventral striatum, amygdala, ventral tegmental area, and midbrain (10). The salience network serves as the “dynamic switch,” biasing activation of other task-positive or task-negative networks when a salient external event is detected (3). Of the salience network regions, the most prominent structures are the insula and anterior cingulate cortex, which consistently show coactivation in response to both internal and external salience (11).

While the posterior insula plays a key role in integrating sensory and motor information to mediate behavioral responses to interoceptive and external cues (12, 13), the anterior portion of the insula functions to integrate this sensory and interoceptive feedback from the posterior insula with cognitive and emotional responses to the same stimuli, to create a conscious evaluation of affective experience (11, 14). Lesions incorporating the insula may reduce an individual’s capacity for integrating external stimuli and thus weaken motivated or appropriate emotional responses. Clinically, insular dysfunction can plausibly account for several characteristic signs of psychoses such as deficits in social cognition, decision-making (13, 15), information processing difficulties (16), and psychotic symptoms (17, 18). Reduced insula–anterior cingulate cortex connectivity has been demonstrated in untreated patients with first-episode psychosis (19), suggesting a normalization of this functional coupling *via* dopamine D_2_ receptor antagonism together with serotonin 2A receptor antagonism (20). A recent resting-state fMRI study showed that antipsychotic-induced improvement of psychotic symptoms was accompanied by increased functional connectivity among striatal regions, the anterior cingulate cortex, and the insula (21). Structural imaging studies have repeatedly and consistently detected gray matter abnormalities in these areas in different stages of schizophrenia (22–24). In addition, the cortex of the right insula becomes significantly thinner in patients with schizophrenia (25).

Chronic schizophrenia patients exhibited increased responses of aberrant salience in the striatum, hippocampus, and prefrontal regions compared with healthy controls (26) and lower responses of adaptive salience in the striatum (27, 28), amygdala, hippocampus, and midbrain (27). Adaptive salience refers to a significantly more rapid response in trials with high probability reinforcement relative to trials with low probability reinforcement (7). Moreover, functional connectivity analysis revealed abnormal functional integration within the salience network in early-stage schizophrenia (24).

Abnormal dopamine transmission is a mainstay of theories that aim to explain the neurobiological correlates of positive symptoms in psychosis. Kapur (5) proposed that a hyperdopaminergic state, which had been described in psychosis (29, 30), leads to aberrant assignment of salience (31). A recent multimodal study demonstrated a relationship between presynaptic capacity for dopamine synthesis and aberrant salience-related neural responses, even in individuals at risk for psychosis (32). As antipsychotics tend to block D_2_ receptors in the dopamine pathways of the brain, antipsychotic medication may reduce positive symptoms, by attenuating dopamine-mediated aberrant motivational salience (5). Accordingly, it can be hypothesized that treatment with antipsychotics would dampen adaptive motivational salience. In fact, it has been shown that medicated patients with schizophrenia exhibited reduced adaptive salience compared to healthy controls at a behavioral level (7).

Although the aberrant salience hypothesis of positive symptoms is appealing, it does not explain treatment resistance, as one-third of patients do not respond to non-clozapine antipsychotics (33), despite high levels of D_2_ occupancy (34). The implication is that, for a significant number of patients, the pathophysiological basis of their symptoms involves more than dopaminergic excess. Overall, this suggests that there may be a “non-dopaminergic” subtype of schizophrenia (35). The involvement of glutamatergic mechanisms in schizophrenia has been hypothesized for many years, and the prevailing hypothesis is that there is primary involvement of NMDA receptor dysfunction (36). However, at present there are some inconsistencies, and this has not led to significant advances in treatment. There are two possible explanations for the involvement of both dopamine and glutamate in schizophrenia. One is that they underlie different subtypes of the disorder, in line with the recent findings in treatment resistance (37). The other is an integrated hypothesis, which could explain positive symptoms in terms of presynaptic dopamine, and negative and cognitive symptoms in terms of glutamate.

We have found reduced *adaptive* salience activation in the right insula in antipsychotic-medicated first-episode patients (FEP) compared to healthy controls. In addition, antipsychotic-free FEP exhibited a negative correlation between *adaptive* salience activation in the right insula and hallucination score (38). Unfortunately, we have not found any significant differences in terms of *aberrant* salience in our previous work (38). This could be related to the fact that positive psychotic symptoms in our included groups were not so strong pronounced (38). With this study, we aimed to include psychotic patients with stronger positive psychotic symptoms and to additionally emphasize clinical symptomatology instead of different stages of psychosis. In a cohort of patients with positive psychotic symptoms of different severity, we hypothesized that more severe psychotic symptoms would be related to stronger aberrant salience at a behavioral level. We expected to find higher blood oxygenation level-dependent (BOLD) signals in the insula during aberrant salience in the patients with more pronounced psychotic symptoms than in those with less pronounced psychotic symptoms.

We further focused on evaluating any interaction between cumulative antipsychotic medication taken during the previous year and aberrant salience processing. We hypothesized that there would be a negative correlation between aberrant salience processing and antipsychotic medication.

## Materials and Methods

### Study Population

Male FEP (*n* = 28) were included who fulfilled the operational criteria for “first-episode psychosis” (39) and were recruited at the Psychiatric Outpatient Department, within an Early Detection of Psychosis (FePsy) clinic (40). Nineteen of the 28 FEP were overlapping with Smieskova et al. (41). FEP fulfilled the transition criteria for a psychotic episode, as described by Yung et al., i.e., hallucination ≥4 or unusual thought content, suspiciousness, or conceptual disorganization ≥5 on the Brief Psychiatric Rating Scale (BPRS) (42). Eleven of the FEP were receiving antipsychotic medication. Additionally, we included 14 individuals with chronic psychotic disorders, who had committed a violent criminal offense related to their severe mental disorder and are therefore being treated with stringent drug monitoring in the Department of Forensic Psychiatry at the Psychiatric Clinic of the University of Basel. These patients fulfilled DSM-IV criteria or the Operational Criteria OPCRIT checklist for psychotic and affective illness (43). At the time of scanning, all patients with chronic psychotic disorders were receiving antipsychotic medication.

Cumulative antipsychotic doses during the previous year were recorded at assessment. Doses of antipsychotics were converted into chlorpromazine-equivalent doses (CPZ), as in previous studies (44, 45).

Furthermore, we collected the drug history of antidepressant medication, alcohol, nicotine, and illegal drugs in a semi-structured interview.

We applied the following exclusion criteria to our groups: history of previous psychotic disorder (only for FEP); psychotic symptomatology caused by an “organic” disorder; recent substance abuse according to ICD-10 research criteria; psychotic symptomatology associated with an affective psychosis or a borderline personality disorder; age under 18 years; inadequate knowledge of German; and IQ less than 70.

Ethical approval was obtained from the local ethics committee, Ethikkommission Nordwest- und Zentralschweiz (EKNZ). All participants provided written informed consent and received compensation for participating.

As we were interested in the associations between the severity of positive symptoms and aberrant salience processing, we separated our cohort (*n* = 42) according to positive symptom scores (i.e., a median split of the sum of BPRS items for suspiciousness, hallucinations, and unusual thought content, and conceptual disorganization). The final subgroups consisted of 21 psychotic patients (10 FEP and 11 individuals with chronic psychotic disorders) with less positive symptoms (mean score = 6) and 21 psychotic patients (18 FEP and 3 individuals with chronic psychotic disorders) with more positive symptoms (mean score = 14). The groups were created prior to assessment, but not to scanning. The staff of the MRI scanner was thus blind to group status.

### Salience Attribution Test

Hemodynamic and behavioral responses during aberrant salience processing were assessed with the Salience Attribution Test (SAT), as previously described (7, 32). During the task, the participants responded to a probe after seeing one of four categories of cues (blue animals, red animals, blue household objects, and red household objects), which varied along two dimensions (color and form). Participants received monetary reward in 50% of trials and earned more money for more rapid responses. The probability of reward varied along the cue dimensions (task-relevant dimension, e.g., color – blue stimuli: 87.5% rewarded; red stimuli: 12.5% rewarded), but not for other dimensions (task-irrelevant dimensions, e.g., form – animal and household stimuli: both 50% rewarded). Two experimental sessions (64 trials each) were performed during fMRI. Participants were not informed about the contingencies, which remained the same over blocks, and had to learn them during the task. They were also asked to estimate reward probabilities for each of the 4 stimulus categories after each session (i.e., after 64 trials), using visual analog scales (VAS) ranging from 0 to 100%.

Aberrant salience was defined as the absolute difference in reaction time (implicit) or VAS (explicit) between the two levels of the task-irrelevant stimulus (collapsing across the dimension of task-relevant stimulus). Since aberrant salience is defined as any such deviation, it is always positive.

Participants had to respond rapidly to the presentation of a square. Money was available in 50% of trials, with the likelihood of reward in a given trial being signaled by one of four categories of cues. The cues varied in two different visual dimensions, with one of these cue dimensions being task-relevant. Participants estimated reward probabilities for each cue category, using VAS in percentage.

### Clinical Scales and Cognitive Measures

Subjects were assessed by experienced psychiatrists or psychologists with the BPRS (46, 47) and the Scale for the Assessment of Negative Symptoms (SANS) (44). Current level of functioning was rated with the Global Assessment of Functioning Scale (DSM-III-R 1987). Intelligence quotient was assessed using the Mehrfachwahl-Wortschatz-Test (MWT-B) (48). The number of years spent in full-time education was obtained at interview.

### Behavioral and Clinical Data Analysis

All analyzes were performed using the Statistical Package for Social Science (SPSS) version 19. Clinical, demographic, and behavioral differences between groups were examined with two sample *t*-tests and χ^2^ tests. Brain activation from the significant between-group differences were obtained from the first eigenvariate of a sphere of 5 mm radius around the peak of activation. To examine the association between brain activation in the region with between-group differences and dose of antipsychotic medication, we performed two-tailed Pearson’s correlation analysis. For all tests, *p* < 0.05 was considered significant and 0.05 < *p* < 0.1, a trend toward significance. The Bonferroni correction (at *p* = 0.05) was applied for all *post hoc* tests.

### Magnetic Resonance Imaging Acquisition

Participants were scanned using a whole body 3 T MRI system (Magnetom Verio, Siemens Healthcare, Erlangen, Germany) and a 12-channel phased-array radio-frequency head coil. During the SAT, we acquired T_2_*-weighted echo-planar images (EPIs) with the following parameters: interleaved acquisition mode, 38 axial slices of 3 mm thickness, 0.5 mm interslice gap, field of view 228 mm × 228 mm, and an in-plane resolution of 3 mm × 3 mm. The repetition time was 2.5 s, the echo time 28 ms, and bandwidth was set to 2350 Hz/pixel. The total scan time for both SAT sessions was 20:50 min (with a practice session to adjust speed of appearance of items of 1:45), thereby yielding a total of 2 × 248 volumes. In addition, structural sequences (MPRAGE) were obtained to register functional images. Structural MRI images were additionally screened for gross radiological abnormalities.

### fMRI Procedure

Echo-planar images were analyzed using Statistical Parametric Mapping (SPM8, www.fil.ion.ucl.ac.uk/spm). The first four images in each series were discarded to allow for signal stabilization. During preprocessing, images were realigned and unwarped, spatially normalized to the Montreal Neurological Institute (MNI) space template (including reslicing to 2 mm × 2 mm × 2 mm voxels), and smoothed with a Gaussian kernel at 8 mm × 8 mm × 8 mm full half-width maximum. We first checked the realignment parameters of each individual to identify scans on which sharp movements [bigger than half of the voxel size (1.5 mm) and/or more than 1.5°] had occurred and inspected those scans manually. Corrupted images were excluded and replaced with the average of the neighbor images using ImCalc in SPM8. No subject had more than 10% corrupted images due to movement. Maximum likelihood parameter estimates were calculated at the first level at each voxel, using the general linear model. Our design matrix included an autoregressive AR(1) model of serial correlations and a high-pass filter with a cutoff of 128 s. The onsets of each event were convolved with the SPM synthetic hemodynamic response function and its temporal and dispersion derivatives. The first level design matrix included four cue regressors, an outcome regressor, and its parametric modulation by magnitude of reward (in Swiss Francs); for more details see Roiser et al. (49). Only aberrant contrasts of reward prediction entered the second level analyses to identify the main effect of motivational salience and between-group differences, using the summary statistics approach to random-effects analysis.

A two sample *t*-test at the whole brain level was used to test for group differences on the *aberrant* contrasts of reward prediction. For between-group differences, significance was assessed at a cluster-level threshold of *p* < 0.001 FWE corrected across the whole brain, using a cluster-forming threshold of *p* < 0.001 (uncorrected) (50). In tables and results, we report cluster-level *p* values.

Effects were visualized in the FMRIB Software Library Viewer and labeled using the incorporated atlas tools (i.e., MNI Structural Atlas and the Juelich Histological Atlas).

The primary goal of this study was to assess the relation between symptom severity and functional brain imaging correlates of aberrant salience processing. However, as we also included criminal offenders into our study, we also decided to investigate the impact of forensic background on aberrant salience processing by comparing forensic and FEP.

## Results

### Demographic and Clinical Data and Behavioral Data

Patients with more positive symptoms were compared to those with less positive symptoms; there were no significant differences in age, verbal intelligence, global functioning, or behavioral data (i.e., explicit or implicit aberrant salience). Patients with less positive symptoms had been treated with higher doses of antipsychotics (*p* = 0.032) in the last year before scanning (Table 1). For a description of the sociodemographics and clinical characteristics of the groups, see (forensic and first episode patients) Table S1 in Supplementary Material.

**Table 1:** **Description of sociodemographic and clinical samples: median split by positive psychotic symptoms**.

Psychotic patients with	Less positive symptoms (10 FEP and 11 forensic)	More positive symptoms (18 FEP and 3 forensic)	*p*
Positive psychotic symptoms^a^	6 ± 1	14 ± 1	<0.0001
Age	31.57 ± 7.69	29.33 ± 7.80	0.355
MWT	107.65 ± 13.76	106.11 ± 14.29	0.748
CPZ	162,973.29 ± 163,944.46	60,926 ± 131,687.29	0.032
GAF	54.76 ± 16.55	49.10 ± 13.57	0.239
**SAT: behavioral data**			
Mean reaction time (ms)	262.10 ± 46.44	260.73 ± 52.42	0.929
Total omissions	0.57 ± 1.36	2.48 ± 6.57	0.201
Premature responses	5.86 ± 8.77	4.33 ± 4.29	0.479
Implicit aberrant salience (ms)	15.79 ± 10.76	15.10 ± 8.52	0.819
Explicit aberrant salience (VAS%)	12.08 ± 11.46	10.89 ± 8.31	0.702

*Data are presented as mean ± SD*.

*^a^Sum of BPRS items (suspiciousness, hallucinations, unusual thought content, and conceptual disorganization)*.

*CPZ, cumulative chlorpromazine equivalents during the previous year; GAF, global assessment of functioning; MWT, intelligence quotient test (Mehrfachwahl-Wortschatz-Intelligenz-Test); SAT, Salience Attribution Test*.

### fMRI Data

**Figure 1:**
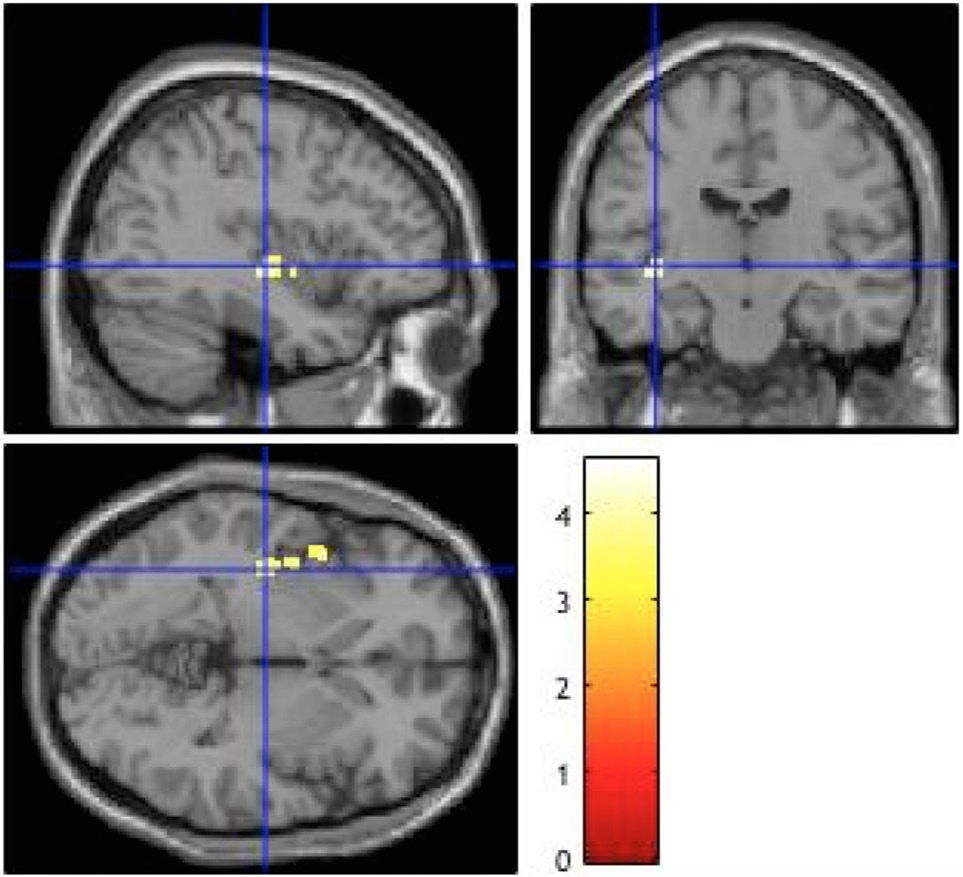
**Brain activation during aberrant salience associated with positive symptoms**. Patients with more positive symptoms compared to those with less positive symptoms showed higher activation in the left posterior insula (*p* = 0.038), The image is displayed at a cluster-level threshold of *p* < 0.001 FWE corrected across the whole brain, using a cluster-forming threshold of *p* < 0.001 (uncorrected), and the right side of the brain is displayed on the right side of the figure.

Compared to the patients with less positive symptoms, patients with more positive symptoms showed a cluster of significantly increased BOLD signals in the left posterior insula (according to the Juelich Histological Atlas) (51) [peak voxel (*x* = −38, *y* = −16, *z* = −2), voxel size 272, *p* = 0.038] (Table 2; Figure 1). Including explicit (*p* = 0.05) and implicit (*p* = 0.049) aberrant salience as a covariate did not alter this result. There were no significant differences between forensic patients and first episode patients without a history of direct violence.

**Table 2:** **Regional brain activations identified by aberrant salience**.

Contrast	Cluster level		Voxel level		*Z*	
	*P*_FWE_-corrected	kE	*P*_FWE_-corrected	MNI		
LowPos < HighPos	0.038	272	0.342	−38 −16 −2	4.10	Left posterior insula
			0.491	−50 −5 4	3.96	Left frontal lobe
			0.961	−46 2 2	3.44	Left frontal lobe
LowPos > HighPos	n.s.					

*LowPos, patients with less positive symptoms; MorePos, patients with more positive symptoms*.

*The upper part of the table presents significant brain regions; patients with less positive symptoms had less activation than patients with more positive symptoms, at a cluster-level threshold of *p* < 0.001 FWE corrected across the whole brain, using a cluster-forming threshold of *p* < 0.001 (uncorrected), and a minimal cluster size of 20 voxel; MNI, coordinates *x y z* according to the Montreal Neurological Institute*.

### Correlations

**Figure 2:**
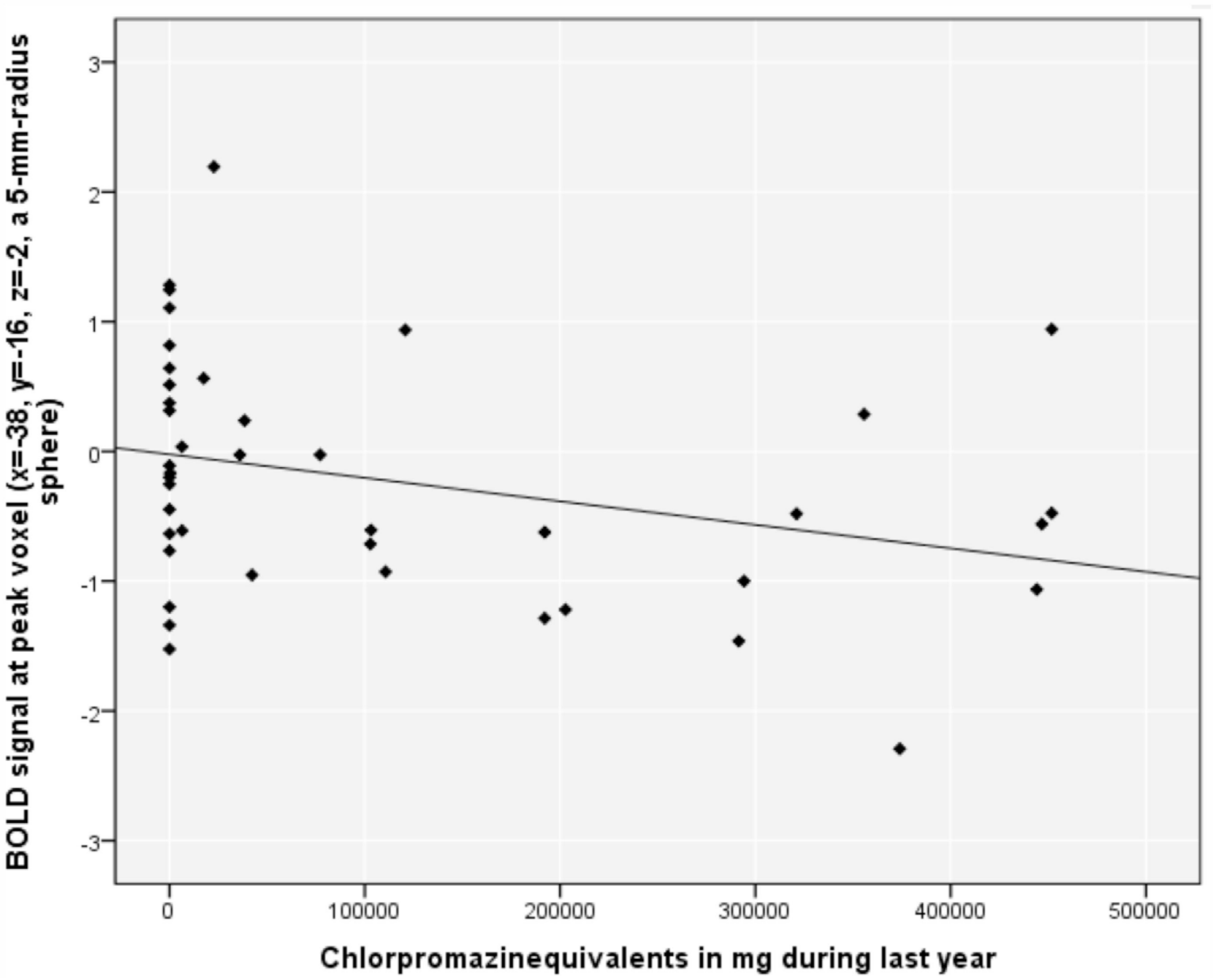
**Relationship between blood oxygenation level-dependent (BOLD) signal in the left posterior insula and chlorpromazine equivalents in milligram during the previous year in psychotic patients**.

Aberrant reward prediction signals in the left posterior insula (time-series extraction at peak voxel from the above analysis in all included subjects in VOI with radius 5 mm) correlated negatively with the CPZ during the previous year (*r* = −0.307, *p* = 0.048) (Figure 2); this correlation did not remain significant after removal of the 17 patients with CPZ = 0 (*r* = −0.308, *p* = 0.134). There was no significant correlation between positive symptoms and implicit (*r* = 0.11, *p* = 0.49) and explicit aberrant salience (*r* = 0.07, *p* = 0.67).

## Discussion

Patients with more positive symptoms showed increased hemodynamic responses in the left insula corresponding to aberrant salience, compared to those with less positive symptoms. In addition, left insula activation correlated negatively with cumulative antipsychotic medication; this effect might be driven by the patients without antipsychotic medication.

In the present study, we focused on psychopathology as a clinical measure, rather than diagnostic group differences. Currently available diagnostic systems (52, 53) seem not to be optimal for classifying the severity of positive symptoms. In addition, it is being increasingly recognized that there is great heterogeneity within psychotic disorders (54, 55).

In contradiction to our first hypothesis, we found no significant behavioral differences with respect to task performance. On the basis of the existing literature, one would expect the patient groups to differ at a behavioral level according to positive symptom severity. However, our results stand in contrast to results from medicated delusional schizophrenia patients [with delusion score 3.2 (SD = 1.0) out of a maximum score of 5], who exhibited significantly greater aberrant salience than those without delusions (7) and to results from clinical high-risk patients who were more likely to attribute motivational salience to irrelevant stimulus features related to the severity of their delusion-like symptoms (32). Our two groups showed almost equal explicit aberrant salience [12.1 (SD = 11.5) and 10.9 (SD = 8.3)], with values similar to those in the high-risk population [12 in Roiser et al. (32)]. The average measure of explicit aberrant salience in HC was 11.2 (SD = 9.1) (49).

This contradiction could be related to the antipsychotic medication: group with less positive symptoms had taken significantly higher doses of antipsychotics during the previous year. Thus, the antipsychotics could have already improved positive psychotic symptoms as well as the measure of aberrant salience, so that there is no behavioral difference between our groups. Another explanation might be that some of our patients with severe delusions belong to the “non-dopaminergic” sub type of psychosis (35), with normal salience but psychotic symptoms due to NMDA receptor dysfunction (36). Another explanation might be that differences in salience are too subtle to be detected in behavior and can only be seen at the functional level.

In partial agreement with our second hypothesis that functional neuroimaging results of aberrant salience relate to positive symptom severity, we found different hemodynamic responses in the left insula. This area is known to have a key role in the salience network (8, 56), as the insula has been shown to be involved in the subjective awareness of internal stimuli (e.g., disgust, anger, sexual arousal) and external stimuli (e.g., pain, taste, temperature) (14, 24). Insular dysfunction in schizophrenia has also been reported to be present during working memory tasks (57), and structural alterations in the right insula seemed to be associated with a higher risk for transition to psychosis in patients with a clinical high-risk state for psychosis (41). Since the salience network has been demonstrated to be critical for integrating internal and external stimuli (3, 58), dysfunction of this circuit in patients with schizophrenia may give rise to the misattribution of experiences, which finally result in hallucinations (59). In psychotic patients, auditory verbal hallucinations evoked increased BOLD signals in the right homolog of Broca’s area, bilateral insula, bilateral supramarginal gyri, and right superior temporal gyrus (60). Another study found that insula activation preceded hallucination onset (61).

In conclusion, our observations are consistent with Kapur’s hypothesis (5), which postulated that inappropriate and excessive salience was responsible for delusions and hallucinations in psychoses.

As the salience network comprises the insula and the anterior cingulate cortex (3, 10, 24, 25, 62–64), we would have expected functional alterations in the latter structure. The absence of alterations in cingular activity is in line with previously published results comparing FEP and healthy controls (38). Our observation might be explained by the normalization of brain function by antipsychotic medication, as the majority of the presented patients were under drug treatment.

As regards the complexity of impairments in psychosis, it is unlikely that clinical manifestations are caused by malfunction of a single brain system (65). It has been discussed whether other brain networks, such as the default mode network including regions in the medial frontal and posterior cingulate cortex, reduce their activity during cognitive demands and may be involved in attention processing (66). Recently, Palaniyappan et al. (9) examined the interactions between the salience system and the central executive network (CEN) and observed a significant failure of both the feedforward and reciprocal influence between the insula and the DLPFC in schizophrenia. These findings provide compelling evidence for a breakdown of the salience–execution loop in the clinical expression of psychosis (9). Reduced cognitive control could lead to reduced attention to salient stimuli, which in turn would then lead to reduced adaptive salience. This reasoning has been supported by studies demonstrating that the severity of cognitive dysfunction in psychosis seems to be influenced by deficits in the CEN (67).

In structural MRI, the association between reality distortion and salience network deficits is based mainly on findings of insular deficits with hallucinations (68–74). A study in monozygotic twins where one sibling suffered from schizophrenia suggested that schizophrenia leads to gray matter loss in the bilateral insular cortex (75). Similar results have been found in populations at high risk for developing schizophrenia (76, 77). In patients with schizophrenia, an inverse relationship has been described between volumes of the right insula and delusions, but not with hallucinations (78).

Previous studies in patients with schizophrenia have found a relationship between left insular volume and positive symptoms (17, 79). Specifically, reductions in left insular volume (74) and activation of this brain area (80, 81) have been associated with auditory hallucinations. Other studies have also reported reductions in the insular cortex specifically associated with paranoid symptoms, as opposed to hallucinations; however, these studies are inconsistent with regard to bilateral versus left insular cortex reductions (82, 83). A multimodal investigation in patients with schizophrenia provided evidence that gray matter reduction in the salience network had functional consequences (25). In particular, the structural defect of the insula was associated with inefficient brain circuit recruitment for information processing.

In addition to structural deficits in salience network, reduced functional connectivity between the midbrain and the insula was found in patients with schizophrenia. The loss in connectivity correlated with increased psychotic symptoms (27). Furthermore, altered between-network interactions during psychotic remission have been demonstrated, which is related to the severity of negative symptoms (56). Altered salience network connectivity with other cortical networks seemed to be a stable characteristic of the pathophysiology of schizophrenia (16, 84), even during the resting state (56, 85).

The relationship between schizophrenia and violence is complex. Among patients with schizophrenia, the risk for violence has been associated with several factors, such as personality traits, substance abuse, intelligence, presence of psychotic symptoms, and demographic background (86). To the best of our knowledge, there is only one study assessing salience in forensic patients: greater impairment in affect recognition performance combined with higher salience and arousal may contribute to the occurrence of violent acts in schizophrenic patients (87). Additionally, it has been demonstrated that positive symptoms in schizophrenic patients can be associated with a criminal history even before diagnosis of a first episode (88). In the present data, there was no difference in BOLD signals between forensic and non-forensic patients, which indicates that the history of a violent criminal offense does not have an important impact on salience processing.

Some limitations of our study merit comment. The sample sizes were modest, and the fact that the majority of our patients were receiving medication may have confounded our findings. Thus, studies exploring antipsychotic-naïve patients are warranted. As antipsychotic medication was very heterogeneous, studies in patients with uniform medication are needed, especially given that atypical and typical antipsychotics may differentially affect brain functioning.

Furthermore, the SAT has been designed to measure abnormal motivational salience processing and its relation to dopamine dysregulation in the VS. However, motivation is not the only form of salience (1), and it would be important to test striatal activation in psychosis during other forms of salience processing that are not measured using speeded response tasks. Further studies should include a healthy control group. As a similar insular activation in the healthy control group and the group with lower scores of positive symptoms would strengthen the evidence for insular dysfunction associated with positive symptoms. Finally, one might argue that the correlation between insular activation and CPZ might simply reflect group differences as CPZ values differ between the two groups.

We conclude that insular salience processing may be altered in psychoses, depending on the severity of positive symptoms. In addition, our results suggest that antipsychotic medication has a negatively modulating effect on insular functioning.

## Author Contributions

SB, AR-R, AE, and RS designed the study and wrote the protocol. CS, RS, AS, and AW managed the literature searches, fMRI analyses, and statistical analysis. AW, RS, FH, CL, and TV helped collecting the data. AW wrote the manuscript. CS helped preparing the manuscript. SB, UL, and AR-R critically revised the manuscript and provided infrastructural support. All the authors contributed to and have approved the final manuscript.

## Conflict of Interest Statement

The authors declare that the research was conducted in the absence of any commercial or financial relationships that could be construed as a potential conflict of interest.
