# Supplementary material for: Altered Insular Function during Aberrant Salience Processing in Relation to the Severity of Psychotic Symptoms
Source: Front Psychiatry. 2016 Nov 23;7:189. doi: 10.3389/fpsyt.2016.00189 (PMC5120113; doi:10.3389/fpsyt.2016.00189)
Supplement: Supplementary file 1 [file Table_1.DOC]

Supplement

**Table 1. Description of sociodemographic and clinical samples by group (forensic and first episode patients)**

|  | Forensic  (N = 14, m = 14) | First episode  (N = 28, m = 28) | p |
| --- | --- | --- | --- |
| Age (years) | 35.29 ± 7.22 | 28.04 ± 6.90 | 0.003 |
| MWT | 107.27 ± 16.01 | 106.67 ± 13.11 | 0.906 |
| CPZ | 303489.89 ± 123410.70 | 16179.86 ± 30609.32 | <0.001 |
| GAF | 41.43 ± 7.78 | 57.48 ± 15.37 | 0.001 |
| Positive psychotic symptoms* | 9 ± 5 | 11 ± 5 | 0.181 |
|  |  |  |  |
| **SAT:**  **Behavioural data** |  |  |  |
| Mean reaction time | 274.77 ± 59.71 | 254.73 ± 42.17 | 0.215 |
| Total omissions | 0.86 ± 1.17 | 1.86 ± 5.81 | 0.53 |
| Premature responses | 6.43 ± 10.17 | 4.43 ± 4.52 | 0.38 |
| Implicit aberrant salience (ms) | 18.72 ± 12.09 | 13.82 ± 7.81 | 0.12 |
| Explicit aberrant salience (VAS %) | 12.77 ± 13.08 | 10.85 ± 8.09 | 0.56 |
