# Supplementary material for: Altered Insular Function during Aberrant Salience Processing in Relation to the Severity of Psychotic Symptoms
Source: Front Psychiatry. 2016 Nov 23;7:189. doi: 10.3389/fpsyt.2016.00189 (PMC5120113; doi:10.3389/fpsyt.2016.00189)
Supplement: Supplementary file 2 [file Table_2.DOC]

Supplement

**Table 2. Distribution of positive symptoms.**

| Positive psychotic symtoms* | N total (FEP/forensic) |
| --- | --- |
| 4 | 6 (4/2) |
| 5 | 2 (0/2) |
| 6 | 5 (4/1) |
| 7 | 4 (0/3) |
| 8 | 4 (0/3) |
| 9 | 2 (2/0) |
| 10 | 4 (4/0) |
| 11 | 1 (1/0) |
| 12 | 2 (0/1) |
| 13 | 1 (1/0) |
| 14 | 4 (4/0) |
| 15 | 2 (0/1) |
| 16 | 1 (1/0) |
| 17 | 1 (1/0) |
| 18 | 1 (1/0) |
| 19 | 1 (1/0) |
| 21 | 1 (0/1) |

***Sum of BPRS items (suspiciousness, hallucinations, unusual thought content, and conceptual disorganisation)**
